# Predicting how color and shape combine in the human visual system to direct attention

**DOI:** 10.1038/s41598-019-56238-9

**Published:** 2019-12-30

**Authors:** Simona Buetti, Jing Xu, Alejandro Lleras

**Affiliations:** University of Illinois, Champaign, United States

**Keywords:** Object vision, Human behaviour

## Abstract

Objects in a scene can be distinct from one another along a multitude of visual attributes, such as color and shape, and the more distinct an object is from its surroundings, the easier it is to find it. However, exactly how this distinctiveness advantage arises in vision is not well understood. Here we studied whether and how visual distinctiveness along different visual attributes (color and shape, assessed in four experiments) combine to determine an object’s overall distinctiveness in a scene. Unidimensional distinctiveness scores were used to predict performance in six separate experiments where a target object differed from distractor objects along both color and shape. Results showed that there is mathematical law determining overall distinctiveness as the simple sum of the distinctiveness scores along each visual attribute. Thus, the brain must compute distinctiveness scores independently for each visual attribute before summing them into the overall score that directs human attention.

## Introduction

Imagine returning from a vacation and having to search for your car (e.g., a dark blue minivan) in a parking lot. What information would the visual system use to help you find it? One possibility is that the feature that is the most discriminative (either color or shape) is used to guide your attention to objects containing that feature. Alternatively, the visual system might use all the discriminative information about the car to find it. Specifically, it might use all the visual attributes (both its color and shape) that make your car different from other cars in the parking lot^1^. Previous work has demonstrated that when the visual difference between a to-be-found object (the target) and its surrounding objects (the distractors) is sufficiently large, human observers can use peripheral vision to compare in parallel the elements in the scene to the target template^2^. This parallel evaluation can be done even when the scene contains different types of distractors, so long as all distractors are sufficiently different from the target^3,4^. In such contexts, the target can be found efficiently without requiring sequential movements of covert or overt attention to inspect individual objects in the scene^5^. That is, although peripheral vision has well-known resolution and processing limitations^6^, when the visual dissimilarity between the target and the distractors is sufficiently large, peripheral vision is able to confidently tell target and distractors apart.

Two questions motivated the present study. First, can search performance in displays containing compound stimuli (defined by shape and color) be predicted based on performance observed in simpler displays, where only one visual feature (color or shape) distinguished the target from distractors? If so, how do the visual differences along distinct feature dimensions combine to predict performance?

**Figure 1:**
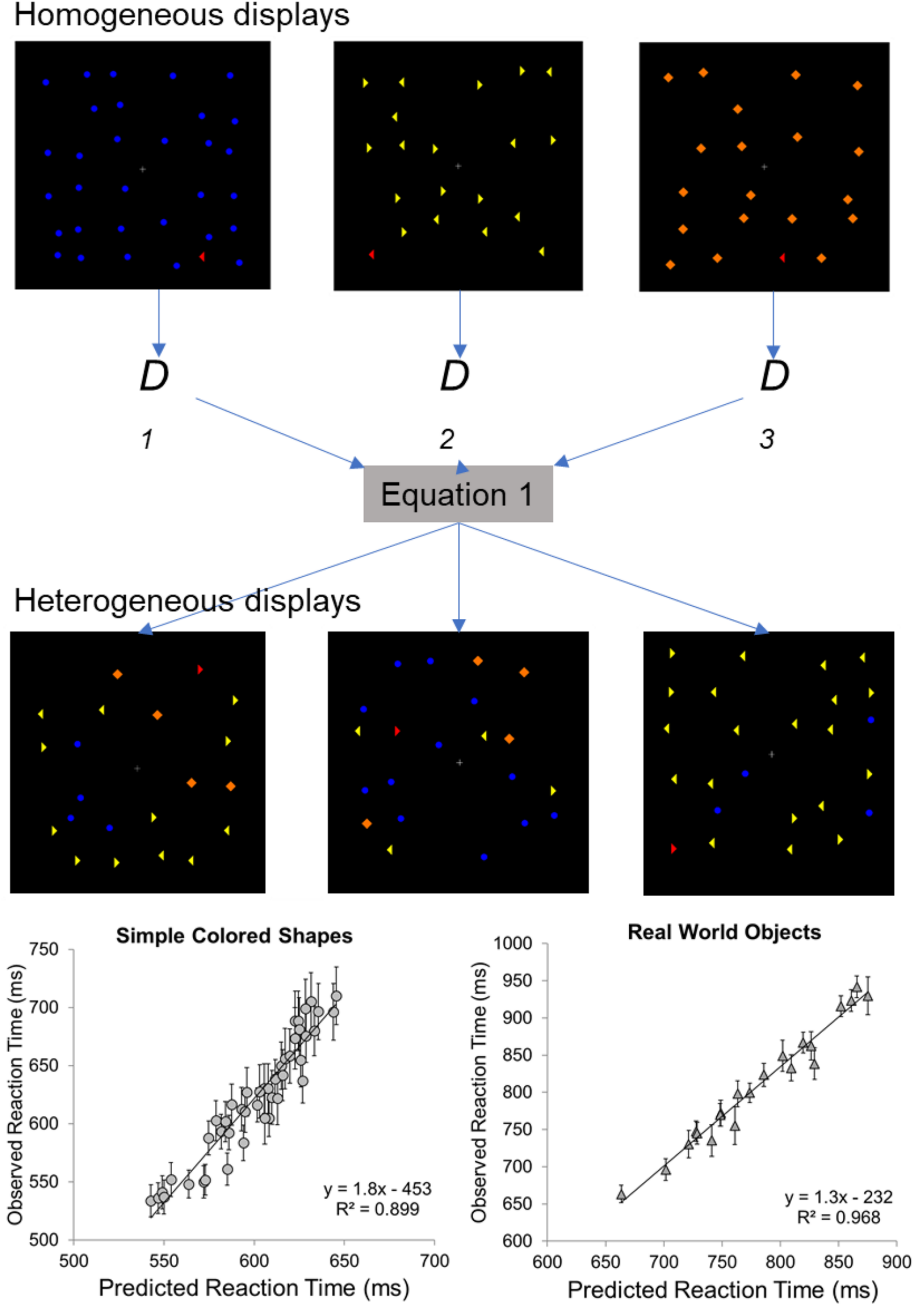
Top panel: Illustration of the approach, using geometric shape stimuli^3^. Performance is first evaluated in homogeneous displays containing only one type of distractor on any given trial. Performance in this task is then used to predict performance in displays simultaneously containing multiple types of items in varying proportions. Bottom panel: Observed Reaction Times in heterogeneous search tasks as a function of predicted Reaction Times based on Eq. 1 when geometric stimuli were tested (left panel, Lleras *et al*.^3^) and when images of real-world objects were used as stimuli (right panel, Wang *et al*.^4^).

**Figure 2:**
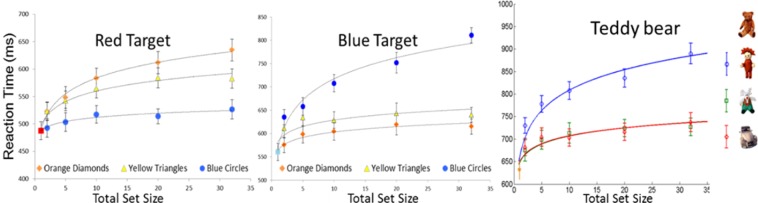
Parallel search efficiency (i.e., logarithmic search slope) varies as a function of target-distractor similarity. The figures show the reaction times when searching for a red triangle among either blue circles, yellow triangles, or orange diamonds (left panel), for a cyan semicircle among the same set of distractors (middle panel), and for a teddy bear among either carrot-top dolls, gray reindeers, or car toys (right panel). High-similarity distractors lead to steeper logarithmic search slopes than low-similarity distractors, indicating longer processing times per item. The left and middle figures have been adapted from Buetti *et al*.^2^ and the right figure from Wang *et al*.^4^.

The present work builds on recent findings from our lab demonstrating that there is systematic variation in reaction times as a function of set size associated with parallel processing^2,4,7–9^ as evaluated in the context of efficient search tasks (see example displays in Fig. 1, top panel). According to the Target Contrast Signal Theory^7^, substantial visual dissimilarity between an item in the scene and the target template in mind allows peripheral vision to easily reject dissimilar distractors in parallel, resulting in reaction times that increase logarithmically as a function of set size. The increase is logarithmic (as opposed to linear) in nature because of the stochastic nature of the parallel evaluation process: some distractors will necessarily be rejected sooner than others, even when they are all identical^10^. The steepness of the logarithmic function is modulated by the similarity between the target and the distractors: more similar distractors lead to steeper logarithmic functions, which reflect longer processing times per item (Fig. 2). This pattern is observed both with simple geometric stimuli that vary along one or two feature dimensions^2^ and with images of complex real-world objects, that vary among a multitude of features^4^.

## Using the Target Contrast Signal Theory to Predict Search Performance in Novel Scenarios

The Target Contrast Signal Theory is a precise mathematical model that allows one to make specific point predictions about how components of visual complexity combine to impact human performance. Specifically, the model estimates the time it takes to evaluate individual items in parallel, with parameters that index target-distractor similarity, number of items present, and distractor homogeneity/heterogeneity. Two recent studies demonstrated that the model can predict with great success search performance in complex heterogeneous scenes based on parameters estimated on simpler homogeneous scenes^3,4^. The approach used in these studies was the following (Fig. 1 top). First, parallel search efficiency (i.e., the logarithmic search slope, Fig. 2) to find a target among different types of distractors was estimated in a group of participants. Importantly, in this first step, displays only contained one distractor type (e.g., either blue circles or orange diamonds or yellow triangles). Second, a new group of participants searched for the same target in heterogeneous displays that contained multiple types of distractors in various combinations (e.g., various numbers of blue circles, orange diamonds, and yellow triangles). The observed reaction times in this last experiment were then compared to the predicted reaction times based on Eq. 1. As shown on Fig. 1 (bottom), when simple geometric shapes were used, Eq. 1 accounted for 89.9% of the RT variance along 45 novel heterogeneous search conditions^3^. When pictures of real-world objects were used, Eq. 1 accounted for 96.8% of the RT variance^4^.

1 $$RT=a+\beta \ast \mathop{\sum }\limits_{j=1}^{L}\,({D}_{j}-{D}_{j-1})\ast \mathrm{ln}\,({N}_{T}-(\mathop{\sum }\limits_{i=1}^{j-1}\,{N}_{i})\ast {1}_{[2,\infty )}(j)+1)$$

In Eq. 1, *L* indicates the number of distractor types present in the display, *N*_*T*_ is the total number of distractors, *N*_*i*_ is the number of distractors of type *i*, and *D*_*j*_ indicates the logarithmic slope parameters associated with distractor of type *j* (organized from smallest *D*_1_ to largest *D*_*L*_). Note that the *D* parameter is the one that increases with increasing target-distractor similarity and these values were estimated in homogeneous displays with a different set of participants. The constant *a* represents the reaction time when the target is alone in the display. Inter-item interactions were indexed by the multiplicative factor *β*. Finally, the index function 1_[2, ∞)_ (j) indicates that the sum over N_i_ only applies when there are at least two different types of lures in the display (j > 1). When j = 1, the second sum is zero.

### Relationship between logarithmic slope, accumulation rate, and contrast signal

The Target Contrast Signal Theory assumes that parallel processing has unlimited capacity: items in the display are simultaneously processed with a rate that does not depend on the number of items. To decide if an item is a non-target, the visual system accumulates information, more specifically, a contrast signal generated by comparing the item to the target template. Accumulation of contrast information is noisy, meaning that at each instant, a different amount of contrast information is sampled from each item. Accumulation rates are a function of a number of parameters like item eccentricity (larger eccentricities have smaller accumulation rates) and lure-target similarity. Importantly, according to the Target Contrast Signal Theory, in homogeneous search conditions, the steepness of the logarithmic slope is inversely proportional to the overall contrast signal between the target and the lures in the display, such that:

2 $$D=\frac{\theta }{C}$$

In Eq. 2, *D* is the value of the observed logarithmic slope in the RT by set size function; *C* the value of the overall contrast signal between target template and distractor stimulus (an inverse of similarity). *θ* is a multiplicative constant. For a given level of dissimilarity (i.e., for each distractor type), the average speed of accumulation is constant, with moment-to-moment stochastic variation. The theory proposes that the accumulation rate is determined by the overall magnitude of the contrast signal between the target template and the distractor being evaluated: more dissimilar distractors (which produce larger contrast signals) will have larger accumulation rates than less dissimilar distractors (which produce smaller contrast signals). Imagine the case of a distractor that is very dissimilar from the target, during the evidence accumulation process, every sample will produce a large amount of evidence that the distractor is not the target, so the evidence will accumulate rapidly and the distractor will be rejected as a non-target very quickly. When the target-distractor distance is not as stark, every sample will provide a smaller amount of evidence that the distractor is not the target, so, evidence will accumulate slower and the decision will take longer. When accumulated evidence reaches a certain threshold, the visual system can confidently conclude that the information at the item’s location is sufficiently different from the target. This location will be rejected in parallel and will not be considered for scrutiny by eye movements. Thus, this threshold represents the visual system’s ability to distinguish items from the target in parallel across the visual field. Two things are worth mentioning. First, the target-contrast signal is not a bottom up signal in the sense that the contrast signal being accumulated is not a contrast between the item and its immediate surroundings. Rather, it is a difference signal between the item being evaluated and the description of the target held in memory. Second, the Target-Contrast Signal theory focuses on understanding the parallel peripheral evaluation and rejection of *lure* stimuli (i.e., distractors that are visually dissimilar from the target, with a signature *logarithmic* increase in reaction times as a function of set size). There are many instances of search where reaction times increase *linearly* as a function of distractor set size, when distractors are relatively similar to the target. TCS proposes that this linear component arises from the serial deployment of attention (or eye movements) to the locations in the scene that parallel peripheral evaluation was unable to discard as containing non-targets. That said, in the current paper, we will focus solely on the parallel search.

### Predictive approach for compound color-shape stimuli

**Figure 3:**
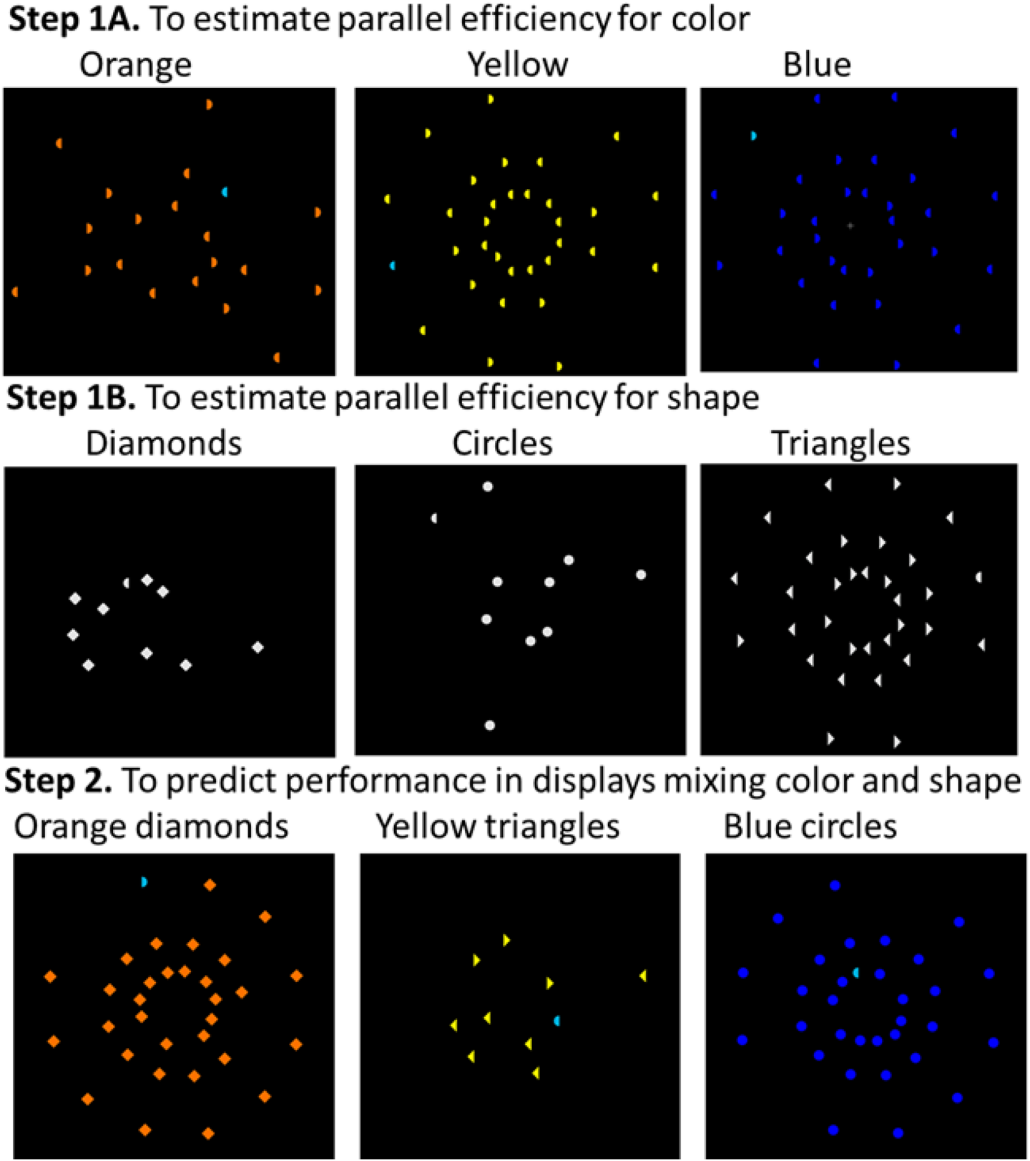
Illustration of the approach used in the present study. In Step 1A, parallel search efficiency was evaluated when participants reported the direction of the cyan semicircle, that was presented among 0, 1, 4, 9, 19, or 31 homogeneous distractors (orange, yellow, or blue) of identical shape. In Step 1B, parallel efficiency was evaluated when participants reported the direction of the gray semicircle, that was presented among a set of 0, 1, 4, 9, 19, or 31 homogeneous gray distractors (either diamonds, circles, or triangles). Steps 1A and 1B illustrate the experimental conditions used in Experiments 1A and 1B. In Step 2, parallel efficiency was evaluated when participants searched targets in displays that contained combinations of features from Steps 1 and 2 (compound stimuli as used in Experiment 2A–C).

Figure 3 provides an illustration of the approach used in the present study. We used homogeneous display throughout the study. Experiments 1 and 2 were conducted first, followed by Experiments 3 and 4 as replication with the same study design but different sets of stimuli.

The first step consisted in estimating parallel search efficiency (i.e., logarithmic search slope) for targets that differ from distractors along a single feature dimension. To estimate parallel search efficiency for specific target-distractors *color* pairings, participants searched for the cyan semicircle target (Experiment 1A) or the red triangle target (Experiment 3A) among orange, yellow or blue distractors. The target and distractors all had the same shape (semicircle in Experiment 1A and triangle in Experiment 3A). To estimate parallel search efficiency for specific target-distractors *shape* pairings, participants searched for the gray semicircle target among diamond-, circle- or triangle-shaped gray distractors (Experiment 1B) or for the gray triangle target among diamond-, circle- or semicircle-shaped distractors (Experiment 3B). Note that the target and distractors all had the same color (gray), when the target was defined by shape (Experiments 1B and 3B), and the target and distractors all had the same shape when the target was defined by color (Experiments 1A and 3A). Table 1 shows the different experimental conditions. Overall, each distractor had an almost reverse similarity relationship to the two possible targets, which allowed us to control for low-level perceptual confounds.

**Table 1:** Logarithmic slopes (ms/log unit of set size) and conditions tested in Experiments 1A-1B and 3A-3B, where target and lures differed along either the color (Experiments 1A and 3A) or shape (Experiments 1B and 3B) dimensions.

*Color efficiency*	*Lure color*	*Lure shape*	*D*
*Experiment 1A* (cyan semicircle target)	Yellow	Semicircle	16.0
	Orange	Semicircle	9.8
	Blue	Semicircle	76.8
*Experiment 3A* (red triangle target)	Yellow	Triangle	22.4
	Orange	Triangle	51.0
	Blue	Triangle	15.5
***Shape Efficiency***			
*Experiment 1B* (gray semicircle target)	Gray	Triangle	141.1
	Gray	Diamond	77.2
	Gray	Circle	62.1
*Experiment 3B* (gray triangle target)	Gray	Semicircle	152.7
	Gray	Diamond	59.3
	Gray	Circle	23.7

*D* indicates the logarithmic slope in the corresponding conditions.

Second, parallel efficiency was evaluated in six new groups of participants who searched for targets in displays where the target differed from distractors along both color and shape (Experiments 2A–C, and Experiments 4A–C). Across the two target templates, there was a total of 18 possible combined feature contrasts (i.e., 2 targets × 3 distractor colors × 3 distractor shapes). Each group of participants searched for a specific target among distractors defined by three possible color-shape combinations. For instance, in Experiment 2A, participants searched for the cyan semicircle among orange diamonds, blue circles, or yellow triangles and in Experiment 2B, participants search for the cyan semicircle among orange circles, yellow diamonds, or blue triangles. Table 2 shows the various combinations tested in each experiment.

**Table 2:** Logarithmic slopes (ms/log unit of set size) and conditions tested in Experiments 2A–C and 4A–C, where the target was presented along with lures that varied in terms of two feature dimensions (color and shape).

*Compound stimuli*	*Lure color*	*Lure shape*	*D*
*Experiment 2A* (cyan semicircle target)	Orange	Diamond	6.9
	Blue	Circle	31.2
	Yellow	Triangle	8.6
*Experiment 2B* (cyan semicircle target)	Orange	Circle	6.8
	Yellow	Diamond	11.7
	Blue	Triangle	55.7
*Experiment 2C* (cyan semicircle target)	Blue	Diamond	44.3
	Yellow	Circle	15.9
	Orange	Triangle	16.7
*Experiment 4A* (red triangle target)	Orange	Diamond	21.4
	Blue	Circle	4.2
	Yellow	Semicircle	18.3
*Experiment 4B* (red triangle target)	Orange	Circle	15.0
	Yellow	Diamond	12.5
	Blue	Semicircle	16.5
*Experiment 4C* (red triangle target)	Blue	Diamond	12.8
	Yellow	Circle	11.0
	Orange	Semicircle	34.2

*D* indicates the logarithmic slope in the corresponding conditions.

Third, the logarithmic slopes observed in the different conditions of Experiments 1A-1B and 3A,B were then used to estimate the predicted logarithmic slopes, using the three models below. Note that all models contain the same number of parameters – two.

#### Best feature guidance model

When the target and lures differ in both color and shape, participants might choose to attend to whichever feature dimension has the strongest discriminability, indexed by the smaller *D* value, i.e., the higher contrast (Eq. 3).

3 $${D}_{overall}=\,{\rm{\min }}\,({D}_{color},{D}_{shape})$$

#### Orthogonal contrast combination model

Given the evidence that color and shape are independently coded in the visual system^11–14^, we hypothesized that feature dimensions compose a multidimensional space where an object can be described by the overall vector in this space. Contrast would be first computed within each feature dimension. In the present case, if the two features are independent, then the magnitude of the overall contrast would be determined by the orthogonal sum of the two within-feature contrast vectors: ${C}_{overall}^{2}={C}_{color}^{2}+{C}_{shape}^{2}$. Because contrast is inversely proportional to *D*, then this formula substitutes to:

4 $${D}_{overall}=\frac{1}{\sqrt{\frac{1}{{({D}_{color})}^{2}+{({D}_{shape})}^{2}}}}.$$

#### Collinear contrast integration model

Whereas the two previous models were determined a-priori at the onset of the project, this third model was discovered after data from Experiments 1 and 2 were analyzed. This model also assumes independence of contrasts along both dimensions, but here, the contrast vectors are not orthogonal to each other but rather collinear. That is to say, whereas visual features create a multidimensional space, contrast exists in a unidimensional space^15^. Thus, the overall contrast is simply the sum of the contrasts from the two feature vectors separately: *C*_*overall*_ = *C*_*color*_ + *C*_*shape*_. The overall *D* being the inverse of the overall contrast, this formula substitutes to:

5 $$\frac{1}{{D}_{overall}}=\frac{1}{{D}_{color}}+\frac{1}{{D}_{shape}}.$$

Fourth, the predicted logarithmic slopes (and predicted response times) from the three models were then compared to the observed logarithmic slopes (and observed response times) from Experiments 2A–C and 4A–C.

## Results

The data and code from this project are publicly available on OSF (https://osf.io/f3m24/).

### Estimating unidimensional discriminability for color and shape

Target and lures varied along one dimension: either color (Experiments 1A and 3A) or shape (Experiments 1B and 3B). The logarithmic slopes from Experiments 1A-1B (semicircle target) and 3A-3B (triangle target) are shown in Table 1. The reaction times as a function of set size and lure type are shown in Fig. S1 (Supplemental Materials).

### Estimating multidimensional discriminability

The lures used in Experiments 2A–C and 4A–C were the result of all the possible color-shape combinations tested in Experiments 1A-1B and 3A-3B, respectively. The logarithmic slopes from Experiments 2A–C (cyan semicircle target) and 4A–C (red triangle target) are shown in Table 2. The reaction times as a function of set size and lure type are shown in Fig. S2 (Supplemental Materials).

### Predictive approach

**Figure 4:**
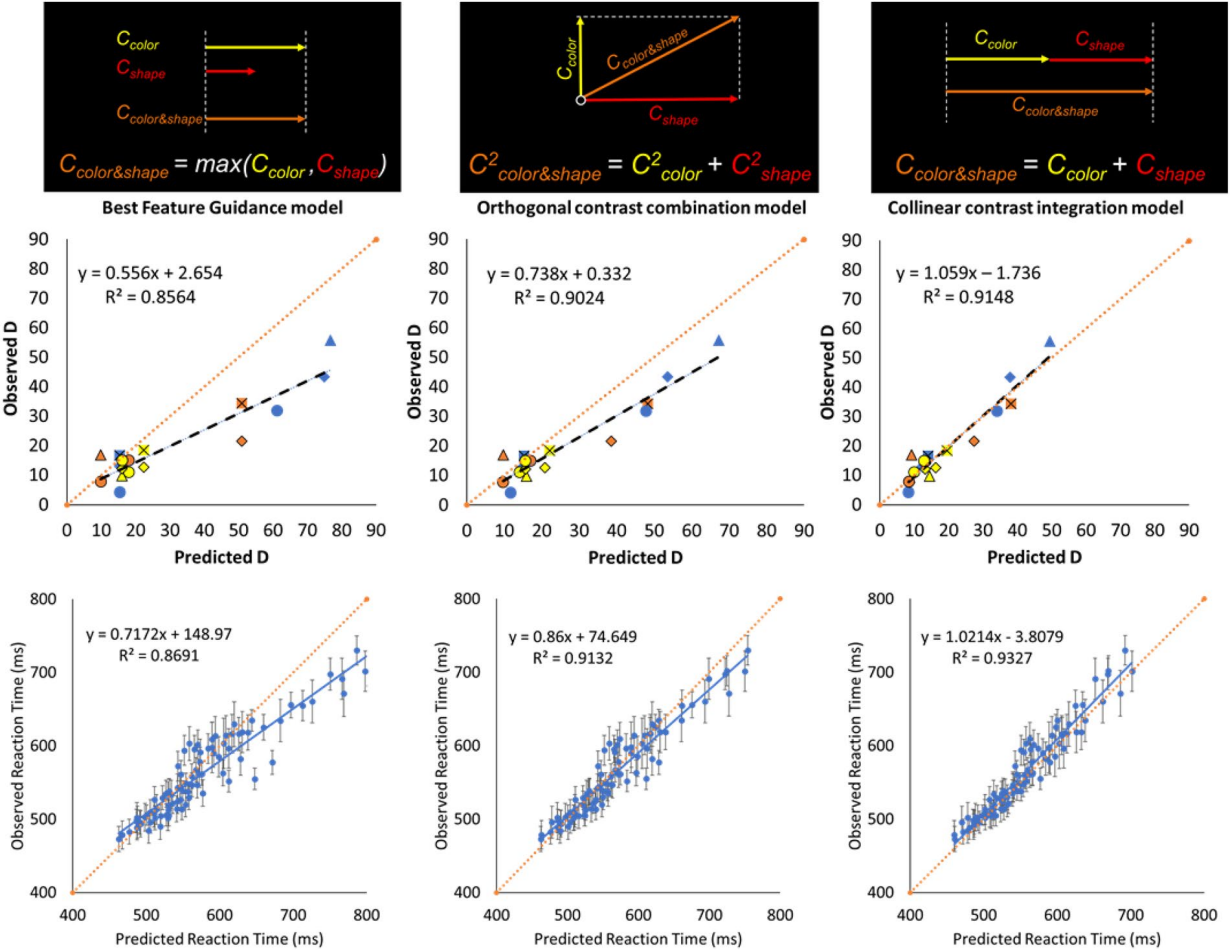
*Top*: Observed search slopes (*D* parameters, Experiments 2A–C and 4A–C) plotted against the predicted search slopes for the three models considered. The dotted line, y = x, is also plotted for reference as it indicates a perfect prediction. Symbols in the figure closely represent the distractor types in the different experiments and are detailed in the Supplementary Text. *Bottom*: The left, middle, and right figures show the observed reaction times plotted against the predicted reaction times for the Best feature guidance model, the Orthogonal contrast integration model, and the Collinear contrast integration model, respectively. Error bars on each data point indicate the standard error of the observed reaction time for each specific condition.

Figure 4 shows the predictions from the three models, combining the results from Experiments 1–4 (Fig. S3 and Tables S1 and S2 show the fits for Experiments 1–2 and Experiments 3–4 separately).

The R^2^ of the Orthogonal contrast combination model (90.24%) and Collinear contrast integration model (91.48%) were both higher than the R^2^ of the Best Feature Guidance model (85.64%). An AIC model comparison (by computing the relative likelihood using exp((AIC_min_ − AIC_i_)/2)) suggested that both the Orthogonal contrast combination model (AIC = 108.59) and Collinear contrast integration model (AIC = 106.15) were 32.4 and 109.81 times more likely, respectively, than the Best Feature Guidance model (AIC = 115.55).

Importantly, the Collinear contrast integration model provided a more precise prediction than the Orthogonal contrast combination model, as indicated by the slope of the regression line in Fig. 4, which was closest to 1 (1.06 vs. 0.74). To further compare the precision of the models, paired t-tests were conducted on the residuals of these models. The residuals were significantly larger than zero for both the Best Feature Guidance model (mean residual = 10.49 log unit/ms) and the Orthogonal contrast combination model (mean residual = 6.34 log unit/ms), t(17) = 3.89, p = 0.0012, dz = 0.554, and t(17) = 4.24, p < 0.001, dz = 0.399, respectively. In contrast, the residuals were not significantly different from zero for the Collinear contrast integration model (mean residual = 0.58 log unit/ms), meaning that there was very good correspondence between the predicted and observed search slopes, t(17) = 0.60, p = 0.5552, dz = 0.044. Finally, the precision of each model’s predictions was estimated as the mean average prediction error as the absolute difference between observed and predicted slopes. The average deviation in the Collinear contrast integration model (mean deviation = 3.41 log unit/ms) was significantly smaller than the ones in the Best Feature Guidance model (mean deviation = 11.37 log unit/ms), t(17) = 3.50, p = 0.0027, dz = 1.047, and in the Orthogonal contrast combination model (mean deviation = 7.27 log unit/ms), t(17) = 3.75, p = 0.0016, dz = 0.975.

Having accurately predicted the logarithmic slope values (*D* parameters) for the 18 different search conditions in Experiments 2A–C and 4A–C allows us to make specific point predictions along the entire RT by set size function for every possible set size and target-distractor conditions tested across the six bidimensional experiments. Figure 4 (bottom) visualizes the success of these point predictions by plotting the 90 observed data points across all six experiments against the predicted RT for the corresponding conditions (the formula used for this is described in the Supplementary Text). The R^2^ was 93.3%, with a mean average prediction error of 13 ms, which is less than 5% of the prediction range (473 ms–730 ms). 85 of the 90 predicted values fell within the 95% confidence interval for the observed RTs.

## Discussion

### Unidimensional contrasts combine linearly to predict bidimensional compound contrast

The results indicated that the discriminability between target and distractors along each feature dimension contributed independently to the overall bidimensional discriminability. Noteworthy, every experiment had an independent group of naïve subjects, and the predictions were made across subjects and types of stimuli. This success confirms the robustness of the predictive approach used in the present and previous studies^3,4^ and it further confirms the stability and predictive value of logarithmic slopes (*D* parameters) estimated in efficient search tasks.

The finding that color and shape contrasts contribute independently to attentional guidance implies that these contrast values are computed separately from one another. This is consistent with prior evidence showing that at the neural level object features like color and shape are initially encoded independently from one another^11–14^. The findings are also consistent with the observation that certain feature pairings, including color and shape, fail independently from one another in working memory and long-term memory, even when the features belong to the same object^16–20^. Thus, we can conclude that not only do these features dimensions contribute separately to memory performance, they (or more precisely, the contrast along these dimensions) also contribute independently from one another towards guiding attention in a scene (but see limitation section regarding generalizability to other feature dimensions).

### Computing contrast between stimuli and target template

The Target Contrast Signal Theory was the framework that inspired the Orthogonal contrast combination and Collinear contrast integration models tested in this study. At its core, the theory assumes that contrast (and not absolute feature values) is the main currency of the visual system and that these contrast values guide attention. The contrast signal guiding attention is obtained from an active comparison between each item in the world and the target template held in mind. In that sense, parallel, efficient search is not so much driven by automatic low-level computations or by feature discontinuities that are present in the scene, but rather by these actively computed contrast signals. From a neurobiological perspective, it makes sense to base a theory of parallel visual processing on the computation of feature comparisons. Indeed, most of the visual brain is aimed at computing contrasts. For example, most of the color-coding neurons in the early visual system code color contrasts, not specific colors in isolation^21,22^. This color contrast computation forms the basis for the opponent-color system^21,22^ and is present as early as color-coding neurons in the LGN. In fact, visual contrasts are computed even before visual information leaves the eyeball^23^. Contrast is the currency of all lateral inhibition signals^24^, of the color system^25^, of boundary detection^26^, and perhaps even of visual categorization^27^.

### Application to models of attention

Several models of visual attention assume that an attended feature receives preferential treatment during processing, such that, for example, the gain for the attended feature is increased or boosted^28–30^. It has been proposed that one feature^30,31^ or even multiple features^32,33^ can guide attention. The present findings indicate that attention is not guided by single- or multiple-specific feature values. Rather search performance seems to be determined by the linear sum of the target-distractor contrasts along the different feature dimensions that differentiate the target from the distractors. This proposal is related to recent findings suggesting that the visual system is actively computing a difference signal to guide attention, as proposed by the relational account of attentional guidance^34–39^. A key insight of this account is that attention is not guided by a specific feature value. Rather it proposes attention is guided by a contrast signal along a goal-defined feature axis, in a manner that is somewhat independent of the feature values themselves that are used to compute that difference score. Together with the present results, this emerging literature suggests attention is guided by actively computed contrasts signals rather than by specific feature values. For a more detailed discussion relating the Target-Contrast Signal model to other attention theories, refer to Lleras *et al*.^7^.

### Limitations and constraints on generalizability

The following limitations of the current findings and constraints on generalizability should be considered. First, we do not expect our findings to generalize to feature dimensions that are not independent from each other (“integral” features in Garner’s terminology^40^). In Garner’s terminology, color and shape are separable feature dimensions and as a result, dissimilarity along both of these dimensions is added according to a “city-block” metric. The city-block dissimilarity measure corresponds to our proposed Collinear Contrast Integration model *C*_*overall*_ = *C*_*color*_ + *C*_*shape*,_ if we were to use the Target-Contrast Signal as the measure of the dissimilarity between target and lure proposed by Garner. In contrast, according to Garner, integral dimensions have a measure of dissimilarity that follows a Euclidian distance. The Euclidian distance dissimilarity measure corresponds to the Orthogonal Contrast combination model examined here (${C}_{overall}^{2}={C}_{dimension\,1}^{2}+{C}_{dimension\,2}^{2}$). Thus, one might expect that when varying dissimilarity along integral dimensions, that the Orthogonal Contrast combination model would be a better predictor than the Collinear Contrast Integration model. More experimentation is required on this front. Second, it is unclear whether search efficiency is impacted by the extent to which observers actively ignore one feature dimension and only focus on the other one. When the target differs from distractors along two dimensions, as in Experiments 2 and 4, how would the D parameters change when the participants actively ignore one of the two features? Even if participants chose to ignore one feature dimension, it is possible that the visual system still processes the ignored dimension as it carries information that can help find the target sooner.

Finally, it might also be worth noting that as the magnitude of the contrast value increases, there is bound to be a floor effect in the observed D parameter (the logarithmic slope becomes “flat”). When D values approach zero, the measurement becomes necessarily noisier, so predicting specific small D parameters will be difficult and might require more observations per condition and more subjects to arrive at a good estimate. This is likely to be a problem when computing overall D parameters for distractors differing from the target along more than two feature dimensions.

## Materials and Methods

The methods and experimental protocols were approved by the Institutional Review Board at the University of Illinois, Urbana-Champaign, and are in accordance with the Declaration of Helsinki. We first ran Experiments 1A, 1B, 2A, 2B and 2C, followed by a set of confirmatory experiments: Experiment 3A, 3B, 4A–C.

### Participants

Participants were all undergraduate students enrolled in a Psychology class in the University of Illinois at Urbana Champaign. Previous experiments conducted in our lab have shown that a sample size of 20 participants is sufficient to obtain stable estimate of logarithmic slope with five set sizes^2,8,9,41^. Informed consent was obtained from all participants.

In Experiments 1 and 2, we ran twenty-five participants per experiment and included the first twenty good participants who met our two inclusion criteria: search accuracy higher than 90% and individual average response times smaller than two standard deviations from the group average response time. Four participants from Experiment 1A were excluded because they did not meet the accuracy criterion. Then, one participant from Experiments 1A (group accuracy = 0.95, mean RT = 671.96 ms, sd = 157.60), two from Experiment 1B (group accuracy = 0.98, mean RT = 742.73 ms, sd = 110.04), two from Experiment 2A (group accuracy = 0.99, mean RT = 657.64 ms, sd = 182.46), two from Experiment 2B (group accuracy = 0.98, mean RT = 626.47 ms, sd = 157.26), and one from Experiment 2C (group accuracy = 0.98, mean RT = 603.61 ms, sd = 99.25) were excluded because they did not meet the response time criterion. In Experiments 3 and 4, the same inclusion criteria were used but data collection ended as soon as 20 participants met the inclusion criteria. As a result, in Experiments 3A and 3B, we collected 22 subjects, and in Experiments 4A–C, we collected 22, 21 and 21 subjects, respectively. One participant from Experiment 3A and one from Experiment 4A were excluded because they did not meet the accuracy criterion. One participant from Experiment 3A (group accuracy = 0.98, mean RT = 613.82 ms, sd = 124.11), two participants from Experiment 3B (group accuracy = 0.99, mean RT = 628.69 ms, sd = 84.64), one from Experiment 4A (group accuracy = 0.98, mean RT = 540.06 ms, sd = 95.60), one from Experiment 4B (group accuracy = 0.99, mean RT = 529.67 ms, sd = 103.94), and one from Experiment 4C (group accuracy = 0.98, mean RT = 517.12 ms, sd = 94.62) were excluded because they did not meet the response time criterion.

### Apparatus and stimuli

All stimuli were presented on a 20-inch CRT monitor at a 85 Hz refresh rate and 1024 × 768 resolution. The experiments were programmed on 64 bit Windows7 PCs in Matlab, using the Psychophysics Toolbox extensions. The target and the lures were about 0.833 degrees of visual angle and were randomly assigned to the display based on an invisible 6-by-6 cells rectangular grid occupying the entire 20-inch display (20 degrees of visual angle).

#### Stimuli used to estimate efficiency in color-only experiments

In Experiment 1A, all stimuli were semicircles pointing to the left or right, and the target was cyan (L = 69, a = −28, b = −35). In Experiment 3A, all stimuli were triangles pointing to the left or right, and the target was red (L = 54, a = 81, b = 70). In both experiments, the lures were either blue (L = 29, a = 68, b = −111), yellow (L = 98, a = −16, b = −93), or orange (L = 65, a = 52, b = 73).

#### Stimuli used to estimate efficiency in shape-only experiments

All stimuli were gray (L = 91, a = 0, b = 0). In Experiment 1B, the target was a semicircle and the lures were either circles, triangles, or diamonds. In Experiment 3B the target was a triangle and the lures were either circles, semicircles, or diamonds.

#### Stimuli used in experiments with compound stimuli

In Experiments 2A–C, the target was a cyan semicircle. In Experiment 2A, the lures were orange diamonds, blue circles and yellow triangles. In Experiment 2B, the lures were yellow diamonds, orange circles, and blue triangles. In Experiment 2C, the lures were blue diamonds, yellow circles, and orange triangles. In Experiments 4A-C, the target was a red triangle. In Experiment 4A, the lures were orange diamonds, blue circles and yellow semicircles. In Experiment 4B, the lures were yellow diamonds, orange circles, and blue semicircles. In Experiment 4C, the lures were blue diamonds, yellow circles, and orange semicircles.

### Design

In all Experiments (1A-1B, 2A-2C, 3A-3B, and 4A–C) there was a target-only condition where no lure was presented, and for each type of lure there were five lure set sizes (1, 4, 9, 19 and 31). In each Experiment, three types of lures were tested (Tables 1 and 2). In total, there were 16 conditions that were repeated 40 times for a total of 640 trials.

### Procedure

At the beginning of each trial, a gray fixation cross appeared at the center of the screen on a black background, followed by a search display. Participants were told to search for the target among distractors and report if the semicircle or triangle target pointed to the left or right. The search display remained on the screen until a response was made by the participants or until 5 seconds passed. If participants made an error or did not respond, a short beep was presented. After each trial, there was an inter-trial interval lasting 1.5 seconds. Eye movements were not restricted or monitored. Note that only RTs from correct trials were included in the analyses.

## Supplementary information

Supplementary Information

## Data Availability

The data and code are available on OSF, link: https://osf.io/f3m24/.
