# Publisher Correction: Predicting how color and shape combine in the human visual system to direct attention

**DOI:** 10.1038/s41598-021-87603-2

**Published:** 2021-04-15

**Authors:** Simona Buetti, Jing Xu, Alejandro Lleras

**Affiliations:** University of Illinois, Champaign, United States

Correction to: *Scientific Reports* 10.1038/s41598-019-56238-9, published online 30 December 2019

This Article contains errors in Equation 4, which was incorrectly given as:

$${D}_{overall}=\frac{1}{\sqrt{\frac{1}{{({D}_{color})}^{2}+{({D}_{shape})}^{2}}}}$$

The correct Equation 4 appears below:

$${D}_{overall}= \frac{1}{\sqrt{{\left(\frac{1}{{D}_{color}}\right)}^{2}+{\left(\frac{1}{{D}_{shape}}\right)}^{2}}}$$

The original Article has been corrected.

