# Supplementary material for: Predicting how color and shape combine in the human visual system to direct attention
Source: Sci Rep. 2019 Dec 30;9:20258. doi: 10.1038/s41598-019-56238-9 (PMC6937264; doi:10.1038/s41598-019-56238-9)
Supplement: Supplementary file 1 — Supplementary Information [file 41598_2019_56238_MOESM1_ESM.docx]

Supplementary Materials for

**Predicting how color and shape combine in the human visual system to direct attention**

Simona Buetti*, Jing Xu, & Alejandro Lleras

Correspondence to: [buetti@illinois.edu](mailto:buetti@illinois.edu)

**This PDF file includes:**

Supplementary Text

Figs. S1 to S3

Tables S1 to S2

Supplementary Text

**Figure 4 additional text. Top.** Symbols in the figure closely represent the distractor types in the different experiments, except for the semi-circle distractor represented here by a square with an “x”. Specifically, we used blue circles, yellow triangles, and orange diamonds in Experiment 2A; blue triangles, yellow diamonds, and orange circles in Experiment 2B; blue diamonds, yellow circles, and orange triangles in Experiment 2C; orange diamonds, blue circles, and yellow semi-circles in Experiment 4A; orange circles, yellow diamonds, and blue semi-circles in Experiment 4B; blue diamonds, yellow circles, and orange semi-circles in Experiment 4C. Note that except for triangle and semi-circles, each type of compound distractors was paired with both types of targets, so there are two data points from those 6 type of distractors in each graph.

The formula used to predict reaction times in Figure 4 (bottom) is the following:

RT_predicted_ = RT_target only_ + *D ** ln(Lure set size+1), where RT_target only_ corresponded to the observed reaction time in the target-only condition for each experiment; *D* was the predicted logarithmic slope for the corresponding lure-target condition, for each of the three models.


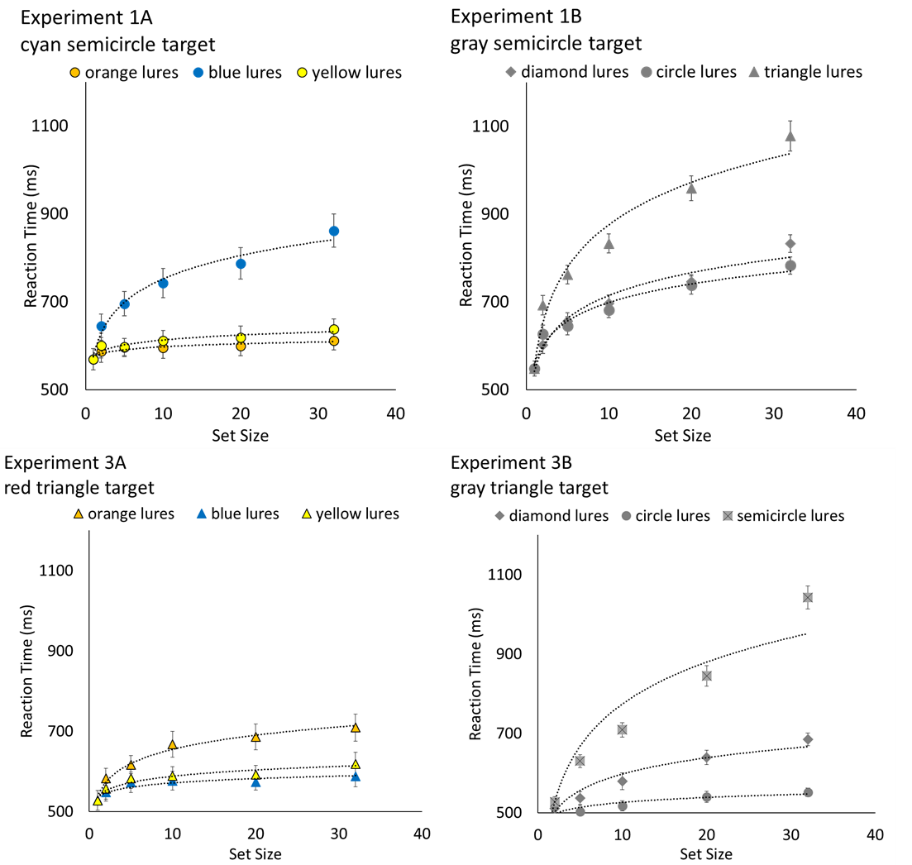


**Fig. S1.** Reaction times from Experiments 1A (top left panel), 1B (top right panel), 3A (bottom left panel), and 3B (bottom right panel) as a function of set size and lure type. Error bars indicate one standard error of the mean.


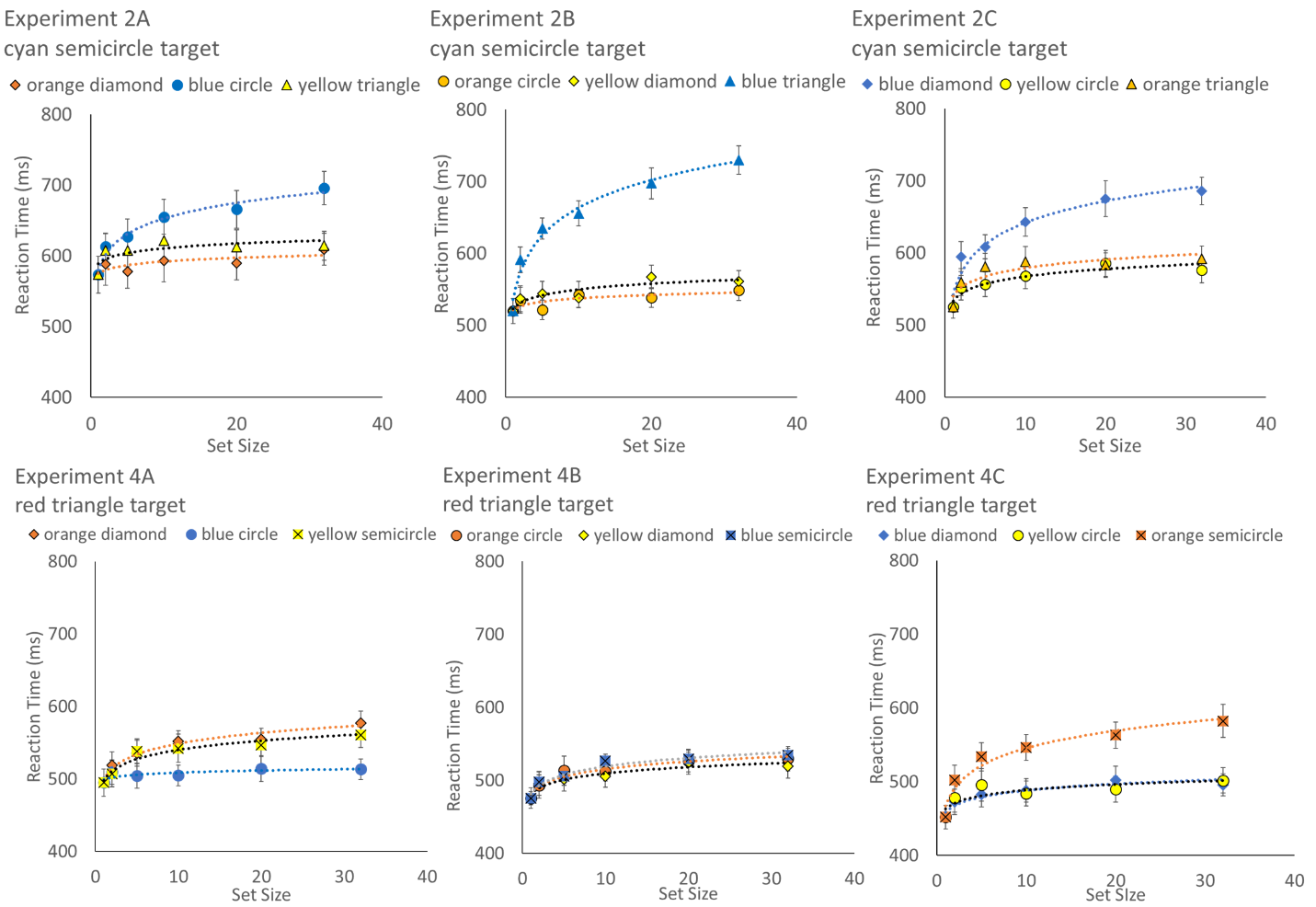


**Fig. S2.** Reaction times from Experiments 2A-C (top) and from Experiments 4A-C (bottom) as a function of lure set size (0, 1, 4, 9, 19, 31) and lure type. Dotted lines show the best logarithmic fit. Error bars indicate one standard error of the mean.


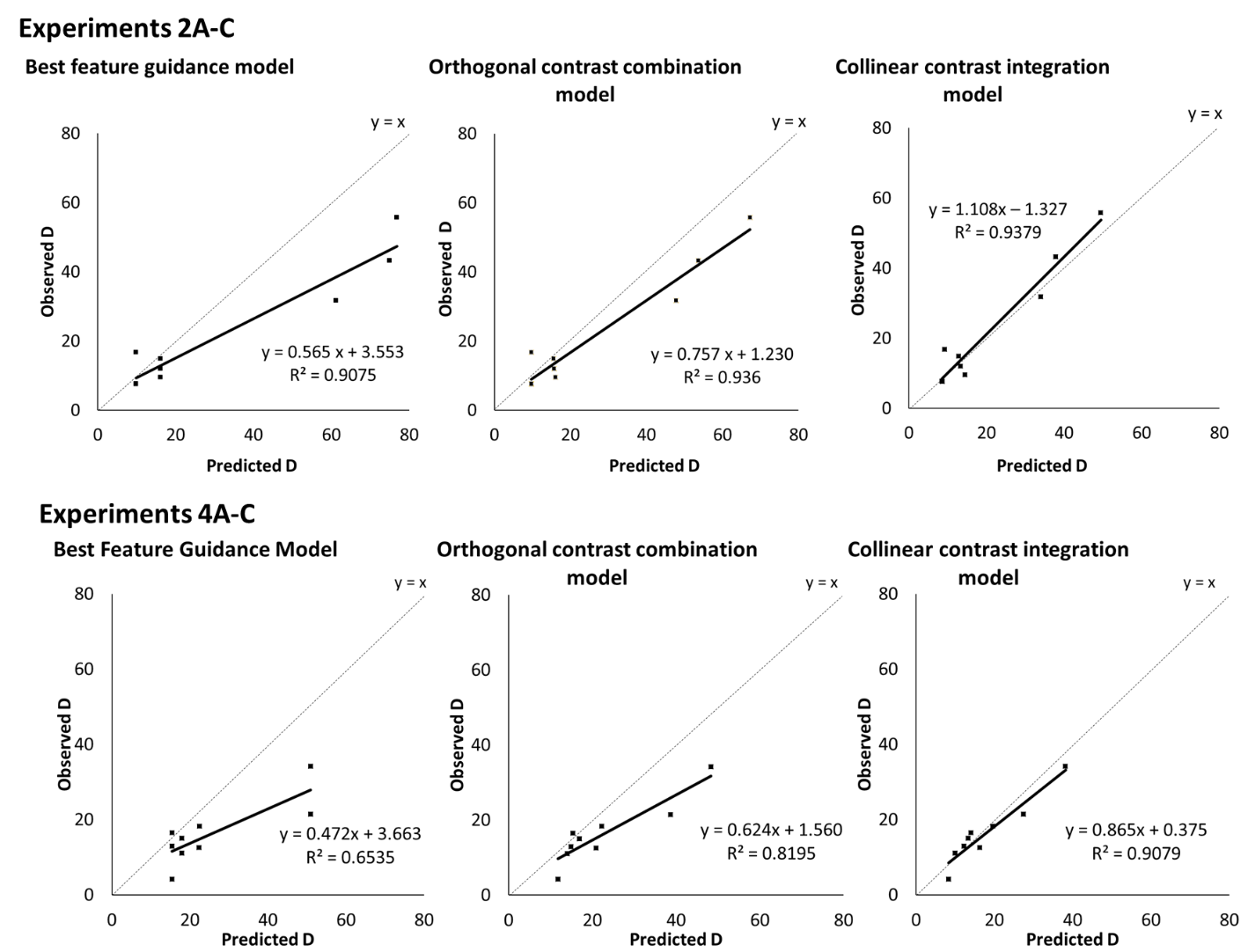


**Fig. S3.** Prediction accuracy for the Best feature guidance model (left panel), the Orthogonal contrast combination model (middle panel) and the Collinear contrast integration model (right panel) based on data from Experiment 2A-C (top) and from Experiments 4A-C (bottom).

**Table S1.** Summary of the three considered regression models, for Experiment 2A-C. Observed search slopes (*D* parameter, Experiment 2A-C) were regressed on the predicted search slopes for the three models considered. The slopes, intercepts and R-squared values are shown in the table for each of the regressions. The Log-likelihood indicates the maximum likelihood estimator for the observed data. AIC estimates the amount of information loss in the model, with smaller AIC values indicating higher quality of a model. The relative likelihood was computed as the exp((AIC_min_-AIC_i_)/2).

| Experiments 2A-C | R-square | log likelihood | Number of parameters | AIC | relative likelihood | Slope | Intercept |
| --- | --- | --- | --- | --- | --- | --- | --- |
| Best feature guidance model | 0.9075 | -27.422 | 2 | 60.843 | 0.167 | 0.565 (0.068) | 3.553 (2.941) |
| Orthogonal contrast combination model | 0.936 | -25.766 | 2 | 57.532 | 0.875 | 0.757 (0.075) | 1.230 (2.603) |
| Collinear contrast integration model | 0.9379 | -25.633 | 2 | 57.266 | - | 1.108 (0.108) | -1.327 (2.763) |

**Table S2.** Summary of the three considered regression models, for Experiment 4A-C. Observed search slopes (D parameter, Experiment 2A-C) were regressed on the predicted search slopes for the three models considered. The slopes, intercepts and R-squared values are shown in the table for each of the regressions. The Log-likelihood indicates the maximum likelihood estimator for the observed data. AIC estimates the amount of information loss in the model, with smaller AIC values indicating higher quality of a model. The relative likelihood was computed as the exp((AIC_min_-AIC_i_)/2).

| Experiments 4A-C | R-square | log likelihood | Number of parameters | AIC | relative likelihood | Slope | Intercept |
| --- | --- | --- | --- | --- | --- | --- | --- |
| Best Feature Guidance model | 0.6535 | -26.524 | 2 | 59.049 | 0.003 | 0.472 (0.130) | 3.663 (3.867) |
| Orthogonal contrast combination model | 0.8195 | -23.591 | 2 | 53.182 | 0.049 | 0.624 (0.111) | 1.560 (2.887) |
| Collinear contrast integration model | 0.9079 | -20.564 | 2 | 47.129 | - | 0.865 (0.104) | 0.375 (2.108) |
